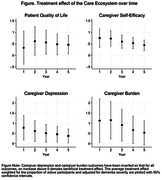# The Long‐Term Effects of the Care Ecosystem Dementia Care Management Program on Quality of Life and Caregiver Well‐being

**DOI:** 10.1002/alz.092994

**Published:** 2025-01-09

**Authors:** Katherine L Possin, Sarah Dulaney, Andrew J Wood, Stephen Bonasera, Isabel Elaine Allen, Alissa Bernstein Sideman, Mia Kanzawa, Jennifer Merrilees, Kirby P Lee, Winston Chiong, Tamara Braley, Sarah M Hooper, Rosalie Gearhart, Helen Bundy Medsger, Krista L Harrison, Lauren Hunt, Rachel Kiekhofer, Chris Chow, Bruce L. Miller, Elan L Guterman

**Affiliations:** ^1^ Memory and Aging Center, UCSF Weill Institute for Neurosciences, University of California, San Francisco, San Francisco, CA USA; ^2^ Global Brain Health Institute, University of California, San Francisco, San Francisco, CA USA; ^3^ University of California San Francisco, San Francisco, CA USA; ^4^ UCSF, San Francisco, CA USA; ^5^ Baystate Health, Springfield, MA USA; ^6^ University of California, San Francisco, San Francisco, CA USA; ^7^ UC Berkeley, Berkeley, CA USA; ^8^ University of Nebraska, Medical Center, Omaha, NE USA; ^9^ University of California Hastings College of the Law, San Francisco, CA USA; ^10^ UC San Francisco, San Francisco, CA USA; ^11^ Facilitator, North Bay Lewy Body Dementia Support Group; Lewy Body Dementia Association, Santa Rosa, CA USA; ^12^ Memory & Aging Center, Department of Neurology, University of California in San Francisco, San Francisco, CA USA

## Abstract

**Background:**

Dementia care management programs, including the Care Ecosystem, have been shown to improve patient and caregiver outcomes, reduce unnecessary healthcare expenditures, and are the focus of Medicare’s new GUIDE payment model. Until now, prior research has focused on evaluating the effectiveness of participating for a short (eg, 12‐month) time frame. The purpose of this study was to evaluate the effects of the Care Ecosystem when delivered for up to 5 years or end of life.

**Methods:**

Of the 804 PLWD‐caregiver dyads enrolled in the previously reported single‐blind 12‐month RCT of the Care Ecosystem (NCT02213458), 456 reported high baseline caregiver burden and were included in this extension trial (NCT04287738). Telephone‐based dementia care management was delivered by a trained care team navigator, with a team of dementia specialists. Primary outcome: PLWD quality of life (QoL‐AD); Secondary: caregiver depression (PHQ‐9), self‐efficacy (Care Ecosystem Self‐Efficacy scale), and burden (Zarit‐12), and PLWD ED and hospital use. The cumulative treatment effect was the sum of the average treatment effect on each outcome at each timepoint, weighted by the number of active participants over time. Linear mixed effects models were used to estimate the treatment effect at the annual timepoints. Caregivers’ perspectives on the value of longer care were derived via semi‐structured interviews after 5 years of participation.

**Results:**

In this prespecified analysis including 297 dyads randomized to Care Ecosystem participation and 159 dyads to usual care, the cumulative 5‐year treatment effect on quality of life, caregiver depression and self‐efficacy were significant; there was a trend for caregiver burden. These treatment effects were most robust during the first 2‐3 years of the study (Figure). The cumulative treatment effects on ED and hospital use were not significant. Most caregivers who participated for the 5 full years indicated that the emotional and practical support was helpful for the duration, but many derived the greatest benefit during the first few years when they were newer to being a caregiver.

**Conclusion:**

The benefits of dementia care management on PLWD quality of life and caregiver well‐being are sustained for five years, with the greatest effects in the first 2‐3 years.